# Effects of bowel cleansing on the composition of the gut microbiota in inflammatory bowel disease patients and healthy controls

**DOI:** 10.1177/17562848231174298

**Published:** 2023-06-06

**Authors:** Péter Bacsur, Mariann Rutka, András Asbóth, Tamás Resál, Kata Szántó, Boldizsár Jójárt, Anita Bálint, Eszter Ari, Walliyulahi Ajibola, Bálint Kintses, Tamás Fehér, Daniella Pigniczki, Renáta Bor, Anna Fábián, József Maléth, Zoltán Szepes, Klaudia Farkas, Tamás Molnár

**Affiliations:** Department of Medicine, Szent-Györgyi Albert Medical School, University of Szeged, Szeged, Hungary; Department of Medicine, Szent-Györgyi Albert Medical School, University of Szeged, Szeged, Hungary; Synthetic and System Biology Unit, Institute of Biochemistry, Biological Research Centre, Eötvös Loránd Research Network (ELKH), Szeged, Hungary; Department of Genetics, ELTE Eötvös Loránd University, Budapest, Hungary; Department of Biochemistry and Molecular Biology, Faculty of Science and Informatics, University of Szeged, Szeged, Hungary; Department of Medicine, Szent-Györgyi Albert Medical School, University of Szeged, Szeged, Hungary; Department of Medicine, Szent-Györgyi Albert Medical School, University of Szeged, Szeged, Hungary; Department of Medicine, Szent-Györgyi Albert Medical School, University of Szeged, Szeged, Hungary; Hungarian Academy of Science – University of Szeged Momentum Epithelial Cell Signaling and Secretion Research Group, Szeged, Hungary; HCEMM-USZ Molecular Gastroenterology Research Group, Szeged, Hungary; Department of Medicine, Szent-Györgyi Albert Medical School, University of Szeged, Szeged, Hungary; Synthetic and System Biology Unit, Institute of Biochemistry, Biological Research Centre, Eötvös Loránd Research Network (ELKH), Szeged, Hungary; Department of Genetics, ELTE Eötvös Loránd University, Budapest, Hungary; HCEMM-BRC Metabolic Systems Biology Research Group, Szeged, Hungary; Synthetic and System Biology Unit, Institute of Biochemistry, Biological Research Centre, Eötvös Loránd Research Network (ELKH), Szeged, Hungary; Doctoral School in Biology, Faculty of Science and Informatics, University of Szeged, Szeged, Hungary; Synthetic and System Biology Unit, Institute of Biochemistry, Biological Research Centre, Eötvös Loránd Research Network (ELKH), Szeged, Hungary; Department of Biochemistry and Molecular Biology, Faculty of Science and Informatics, University of Szeged, Szeged, Hungary; HCEMM-BRC Translational Microbiology Research Group, Szeged, Hungary; National Laboratory of Biotechnology, Biological Research Centre, Eötvös Loránd Research Network (ELKH), Szeged, Hungary; Synthetic and System Biology Unit, Institute of Biochemistry, Biological Research Centre, Eötvös Loránd Research Network (ELKH), Szeged, Hungary; Department of Medicine, Szent-Györgyi Albert Medical School, University of Szeged, Szeged, Hungary; Department of Medicine, Szent-Györgyi Albert Medical School, University of Szeged, Szeged, Hungary; Department of Medicine, Szent-Györgyi Albert Medical School, University of Szeged, Szeged, Hungary; Department of Medicine, Szent-Györgyi Albert Medical School, University of Szeged, Szeged, Hungary; Hungarian Academy of Science – University of Szeged Momentum Epithelial Cell Signaling and Secretion Research Group, Szeged, Hungary; HCEMM-USZ Molecular Gastroenterology Research Group, Szeged, Hungary; Department of Medicine, Szent-Györgyi Albert Medical School, University of Szeged, Szeged, Hungary; Department of Medicine, Szent-Györgyi Albert Medical School, University of Szeged, Szeged, Hungary; Department of Medicine, Albert Szent-Györgyi Medical School, University of Szeged, Kálvária Avenue 57, H-6720 Szeged, Hungary

**Keywords:** bowel cleansing, bowel preparation, gut microbiota, inflammatory bowel disease, sodium picosulfate

## Abstract

**Background:**

In patients with inflammatory bowel disease (IBD), Crohn’s disease (CD), and ulcerative colitis (UC), numerous cases of exacerbations could be observed after colonoscopy, raising the possible pathogenetic effect of colonic microbiota alterations in IBD flare.

**Objectives:**

We aimed to investigate the changes in the fecal microbiota composition in IBD patients influenced by the bowel preparation with sodium picosulfate.

**Design:**

We enrolled patients with IBD undergoing bowel preparation for colonoscopy in the prospective cohort study. The control group (Con) comprised non-IBD patients who underwent colonoscopy. Clinical data, blood, and stool samples were collected before colonoscopy (timepoint A), 3 days later (timepoint B), and 4 weeks later (timepoint C).

**Methods:**

Disease activity and gut microbiota changes were assessed at each timepoint. Fecal microbiota structure – at family level – was determined by sequencing the V4 region of the 16S rRNA gene. Statistical analysis included differential abundance analysis and Mann–Whitney tests.

**Results:**

Forty-one patients (9 CD, 13 UC, and 19 Con) were included. After bowel preparation, alpha diversity was lower in the CD group than in the UC (*p* = 0.01) and Con (*p* = 0.02) groups at timepoint B. Alpha diversity was significantly higher in the UC group than in the CD and Con (*p* = 0.03) groups at timepoint C. Beta diversity difference differed between the IBD and Con (*p* = 0.001) groups. Based on the differential abundance analysis, the Clostridiales family was increased, whereas the *Bifidobacteriaceae* family was decreased in CD patients compared to the Con at timepoint B.

**Conclusions:**

Bowel preparation may change the fecal microbial composition in IBD patients, which may have a potential role in disease exacerbation after bowel cleansing.

**Summary:** Alterations of gut microbiota is associated to inflammatory bowel disease pathogenesis. Flare-ups are observed after bowel cleansing prior to colonoscopy. Our study focused on the short- and long-term effects on colonic microbiota composition following bowel cleansing with sodium picosulfate using healthy control participants.

## Introduction

The gastrointestinal microbiome is composed of more than 1500 species, which are organized into more than 50 strains. Phyla *Bacteroides*, *Firmicutes*, *Actinobacteria*, and *Proteobacteria* account for the largest number of species.^1–5^ The beneficial bacterial colonization of the human gastrointestinal tract has been shown to play a crucial role in intestinal physiology, especially by maintaining the epithelial barrier function of the gut, by hindering pathogen overgrowth and supporting digestion. Chronic intestinal disorders, such as inflammatory bowel disease (IBD) – Crohn’s disease (CD), and ulcerative colitis (UC), are usually associated with consistent compositional shifts in the gut microbiota, which is considered to have an impact on the disease pathogenesis.^6,7^ According to the accepted theory of IBD, disease pathogenesis seems to be partly the consequence of an abnormal immune response induced by luminal antigen exposure. However, it is still unclear whether dysbiosis is the cause or consequence.^8,9^ It is clear that the composition of the gut flora is different in remission and relapse and that there are protective and aggressive strains for IBD.^10,11^

Colonoscopy is not only the most objective tool for dysplasia screening, but also, with the spread of the treat-to-target approach, the most important diagnostic tool in IBD for determining mucosal and histological healing. The majority of investigations in IBD patients, except in cases with severe flare-ups, require a bowel preparation. Bowel preparation prior to colonoscopy is a routine procedure and considered safe.^12^ However, the exacerbation of the complaints could be observed in some cases after colonoscopy in IBD patients. In these patients, the potential mucosal irritation/inflammation caused by bowel preparation has special importance.^13^

The impact of bowel preparation on the microbiome has been investigated in only a few studies with a small number of cases at different timepoints and with different methodologies.^14–17^ Altogether, <70 patients were studied and only five of them suffered from CD and three from UC; therefore, there is insufficient data on the modulating effect of colonoscopic preparation on the gut flora in IBD.

We aimed to investigate the short- and long-term changes of the gut microbiota composition after the bowel preparation using split-dose sodium picosulfate and magnesium oxide before colonoscopy in IBD patients and healthy individuals.

## Materials and methods

### Patients and definitions

IBD patients with dominated colonic location and healthy controls (Con) scheduled for a colonoscopy were prospectively enrolled between April 2019 and January 2021. The reporting of this study conforms to the STROBE statement.^18^ Indication of endoscopy was dysplasia surveillance and check of mucosal activity in IBD and colorectal cancer screening in Con. Bowel preparations were performed with split-dose sodium picosulfate and magnesium oxide (10 mg and 3.5 g per dose) in all cases. We included IBD patients with proved diagnosis and stable pharmacotherapy at least 3 months before colonoscopy, and we excluded individuals who have taken antibiotic or probiotic treatments, gastric acid inhibitors, or nonsteroidal anti-inflammatory drugs within the last 6 weeks before inclusion. We also excluded patients with moderate-to-severe IBD activity, those aged <18 years, pregnant women, and those who withdrew consent. Con participants with non-negative colonoscopy findings were also excluded. Demographic and clinical data including sex, current age, age at diagnosis, disease duration, disease type, disease localization and extension (using Montreal classification^19^), and behavior data (using Montreal classification^19^) were gathered at inclusion before colonoscopy (at timepoint A).

### Sample collection and storage

Blood and stool samples were collected and analyzed 1 week before colonoscopy (timepoint A), at 3 days after colonoscopy (timepoint B), and at 4 weeks after colonoscopy (timepoint C).^14^ Blood tests were performed to measure C-reactive protein (CRP), serum iron, hemoglobin, and hematocrit levels as well as leukocyte and platelet counts immediately after sampling. Stool samples were obtained at each timepoint and stored into 8-mL plastic tubes (Biolab^®^, Budapest, Hungary) without buffer and stored at −20°C until analysis of the microbiota profile, which was performed within 2 weeks after sample collection. Each patient provided an additional stool sample at timepoint A to exclude infectious agents via microbiological testing.

### Definitions

Clinical activity was assessed using CD Activity Index (CDAI; active disease >150)^20^ in case of CD and a clinical subscore of Mayo (pMayo) (active disease >2)^21^ in case of UC, whereas biochemical activity was assessed by measuring the CRP level (activity >5 mg/L). Endoscopic activity was assessed using the Simple Endoscopic Score for Crohn’s Disease (SES-CD, active disease >3),^22^ and Mayo endoscopic subscore (eMayo) (active disease >1).^21^

### Bacterial composition analysis

Fecal microbiota structure was determined by sequencing the V4 hypervariable region of the 16S rRNA genes. The DNA was extracted from defrosted stool samples by using the ZR Fecal DNA MiniPrep™ kit (Zymo Research, Irvine, California, USA) following the instructions of the manufacturer, including bead-beating mechanical lysis . For DNA isolation, we needed a ⩽150 mg fecal sample per patient. After isolation, the samples were stored at −80°C until sequencing. The V4 region of the 16S rRNA gene was amplified with indexed Illumina primer-pairs (i5-i7) using Phusion high-fidelity DNA polymerase (Thermo Scientific, Waltham, Massachusetts, USA). In the polymerase chain reaction (PCR) mixture, the final primer concentration was 0.4 μM and the template DNA was used in 20-ng concentration. PCR products were isolated from the gel and, after purification, sent for dual-index paired-end Illumina MiSeq™ sequencing using 250-bp reads (Illumina Inc., San Diego, CA, USA). The 16S rRNA sequence data were analyzed using the dada2 R package (R Foundation, Vienna, Austria).^23^

We used the Shannon diversity index to calculate the alpha diversity for measuring bacterial richness of a population.^24^ Beta diversity for measuring bacterial differences among populations was calculated with the Bray–Curtis diversity metric in R using the vegan package’s vegdist function with Cailliez correction method. Principal coordinate analysis (PCoA) was performed to visualize the beta diversity comparisons. To explore the differences between groups’ abundance at the bacterial family level, a differential abundance analysis was performed.

### Statistical analysis

Statistical analyses were performed using R statistical software version 4.1.1 (R Foundation, Vienna, Austria). Descriptive statistics were interpreted as mean ± standard deviation or median + interquartile range for continuous variables and counts and percentages for categorical variables. Alpha and beta diversities and differential abundance analysis were calculated using vegan R package.^23,25–27^ Statistical significance of beta diversity comparison was measured by performing the PermANOVA test.^28^ Normality of samples was tested by visual interpretations. Continuous variables were analyzed with Mann–Whitney *U* test or Welch tests for independent samples to compare differences between groups (after assumptions checked in cases of each test), whereas categorical variables were analyzed using chi-squared and Fisher’s exact tests to compare the proportions among groups. Paired *t*-tests were used to compare the clinical and biochemical activities during examination. The Benjamini–Hochberg procedure was applied to reduce statistical bias. *p*-Value of <0.05 was considered statistically significant. Details of statistical power analysis are available in Supplemental Data.

## Results

### Baseline characteristics

Altogether, 41 patients, comprising 13 UC patients, 9 CD patients, and 19 controls, were included in our cohort. The male/female ratios were 46.15%, 33.33%, and 50% for the UC, CD, and Con groups. The median disease duration was 11.06 (13.01) and 5.05 (6.11) years for the UC and CD patients, respectively (*p* = 0.06). In total, 38.46% of the UC patients had proctitis, whereas most of the CD patients had pure colonic disease (44.44%). More than half of the CD patients had an inflammatory phenotype (55.56%). Clinical activities showed remission to mild disease at UC (pMayo 2 ± 2.1) and inactive disease at CD (CDAI 79 ± 51.60) patients. Slightly elevated CRP levels displayed mild disease at both IBD groups (*p* = 0.798). The coupling data are shown in Table 1.

**Table 1.** Baseline clinical and demographic characteristics of the patients.

Characteristics	UC (*N* = 13)	CD (*N* = 9)	*p* Value
Sex, male, *N* (%)	6 (46.15)	3 (33.33)	0.548
Age, median (IQR)	45.54 (12.46)	32.03 (7.59)	0.009
Disease duration, years, median (IQR)	11.06 (13.01)	5.05 (6.11)	0.06
UC disease extension^+^, *N* (%)			
Proctitis	5 (38.46)		
Distal colitis	4 (30.77)		
Pancolitis	4 (30.77)		
CD disease location^+^, *N* (%)			
Ileum		2 (22.22)	
Colon		4 (44.44)	
Ileocolic		3 (33.33)	
Upper GI involvement		2 (22.22)	
CD disease behavior^+^, *N* (%)			
Inflammatory disease		5 (55.56)	
Stricturing disease		4 (44.44)	
Penetrating disease		0 (0)	
CD perianal manifestation, *N* (%)		0 (0)	
Extraintestinal symptoms, *N* (%)			
Arthralgia	3 (23.08)	3 (33.33)	–
Skin disease	3 (23.08)	2 (22.22)	
Eye disease	0 (0)	0 (0)	
Disease activity, mean (SD)			
pMayo	2 (2.10)		–
CDAI		79 (51.60)	
CRP (mg/L)	6.93 (8.15)	7.70 (5.75)	0.798
Treatment, *N* (%)			
Oral 5-ASA	9 (69.23)	1 (11.11)	0.012
Oral corticosteroids	1 (7.69)	2 (22.22)	0.544
Immunosuppressants	6 (46.15)	5 (55.56)	0.999
Biological treatment	7 (53.85)	5 (55.56)	0.999

5-ASA, 5-aminosalicylates; CD, Crohn’s disease; CDAI, Crohn’s Disease Activity Index; CRP, C-reactive protein; IQR, interquartile range; N, number of participants; pMayo, clinical subscore of the Mayo score; SD, standard deviation of the mean; UC, ulcerative colitis.

+ Using Montreal classification.

### Endoscopic examination and disease activity

Most of the patients had mild endoscopic activity (mean eMayo 1.36 ± 1.08 and SES-CD 3.38 ± 3.54) at baseline . Three patients experienced a relapse of IBD with an elevated CRP level (2.6–12.8, 5.2–16.7, and 9.9–94.6 mg/L) after the colonoscopy. CDAI showed remission at both baseline (79 ± 51.6) and after (62 ± 28) the lavage, although the change was not significant (*p* = 0.269). The clinical activity indices of UC patients were mildly elevated at both measurement points (mean 2 ± 2.1 at baseline and 2 ± 3 after lavage, *p* = 0.853). Therapeutic optimization was required in seven patients (four patients with UC and three patients with CD). A new biological agent was administered in three patients (infliximab, *n* = 1; vedolizumab, *n* = 1; ustekinumab, *n* = 1), whereas adalimumab dose intensification was indicated at one patient. Corticosteroid was administered in two cases and conventional treatment was supplemented with local formulation in the remaining case.

### Alpha diversities

**Figure 1.**
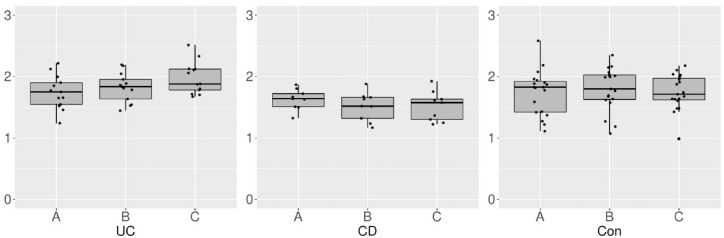
Alpha diversity comparison of groups at different timepoints, visualized as boxplots on a scale of 0–3. Points represent individual samples. Diversity of the ulcerative colitis (UC) group was increased (*p* = 0.04), whereas no difference was found in the Crohn’s disease (CD) and control (Con) groups during this interval (*p* = 0.25 and *p* = 0.81).

**Figure 2.**
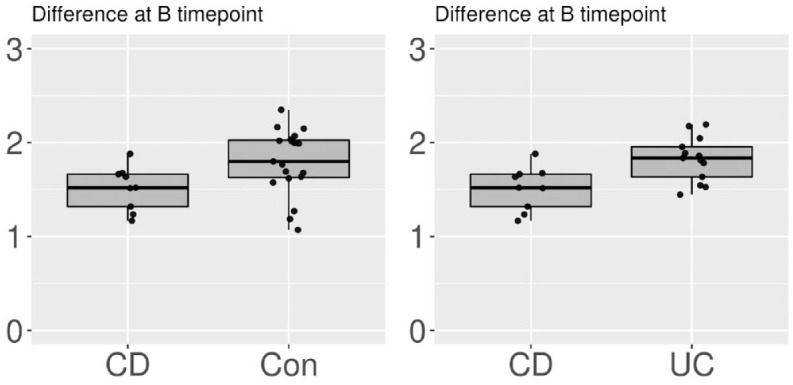
Alpha diversity comparison of groups at timepoint B, visualized as boxplots on a scale of 0–3. Points represent individual samples. At timepoint B, CD patients had the lowest diversity (CD *versus* Con at B *p* = 0.02 and CD *versus* UC *p* = 0.01). CD, Crohn’s disease; UC, ulcerative colitis.

**Figure 3.**
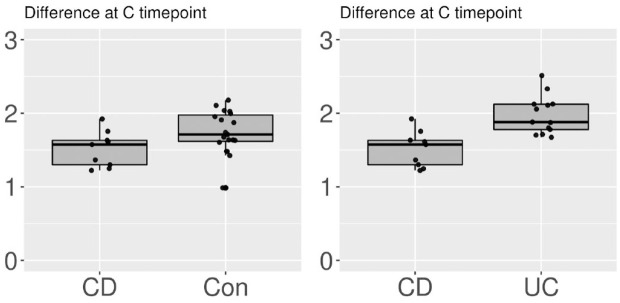
Alpha diversity comparison of groups at timepoint C, visualized as boxplots on a scale of 0–3. Points represent individual samples. At timepoint C, CD patients had the lowest diversity (CD *versus* Con at C *p* = 0.03 and CD *versus* UC *p* < 0.01). CD, Crohn’s disease; UC, ulcerative colitis.

**Figure 4.**
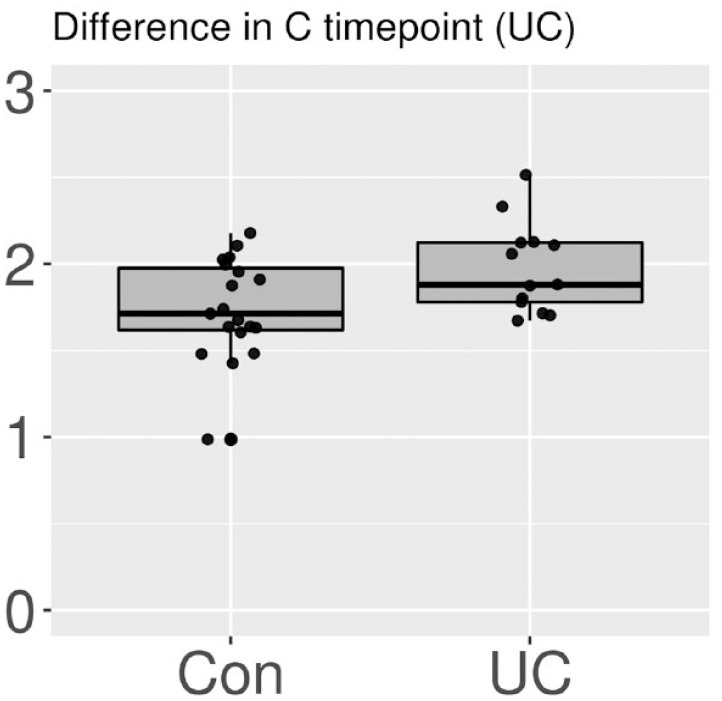
Alpha diversity comparison of the UC and Con groups at timepoint C, visualized as boxplots on a scale of 0–3. Points represent individual samples. At timepoint C, UC patients had a higher diversity compared to the Con (*p* = 0.02). CD, Crohn’s disease; UC, ulcerative colitis.

Although there was no significant difference in the alpha diversities between the three groups at timepoint A, it was increased from timepoint A to C in the UC group (*p* = 0.04). Contrarily, the alpha diversities did not significantly differ between the CD (*p* = 0.25) and Con (*p* = 0.81) groups in this interval (Figure 1). After the bowel preparation, the alpha diversity was lower in the CD group than in the UC (*p* = 0.01) and Con (*p* = 0.02) groups at timepoint B (Figure 2). The alpha diversity was significantly higher in the UC group than in the CD (*p* < 0.01) and Con (*p* = 0.02) groups at timepoint C (Figures 3 and 4). The data on alpha diversity comparisons are shown in Table 2.

**Table 2.** Alpha (Shannon) diversity comparison between groups.

Group	*a*	Group	*a*	*p* Value
CD-A	1.62	Con-A	1.73	0.26
UC-A	1.74	CD-A	1.62	0.29
UC-A	1.74	Con-A	1.73	1.00
CD-B	1.51	Con-B	1.79	0.02
UC-B	1.82	CD-B	1.51	0.01
UC-B	1.82	Con-B	1.79	1.00
CD-C	1.51	Con-C	1.74	0.03
CD-C	1.51	UC-C	1.97	<0.01
UC-C	1.97	Con-C	1.74	0.02
CD-A	1.62	CD-B	1.51	0.38
CD-A	1.62	CD-C	1.51	0.25
CD-B	1.51	CD-C	1.51	1.00
Con-A	1.73	Con-B	1.79	0.58
Con-A	1.73	Con-C	1.74	0.81
Con-B	1.79	Con-C	1.74	0.52
UC-A	1.74	UC-B	1.82	0.51
UC-A	1.74	UC-C	1.97	0.04
UC-B	1.82	UC-C	1.97	0.26

a, Shannon’s alpha; A, B, and C, the time of sampling; CD, Crohn’s disease; Con, control group; UC, ulcerative colitis.

### Beta diversity differences

**Figure 5.**
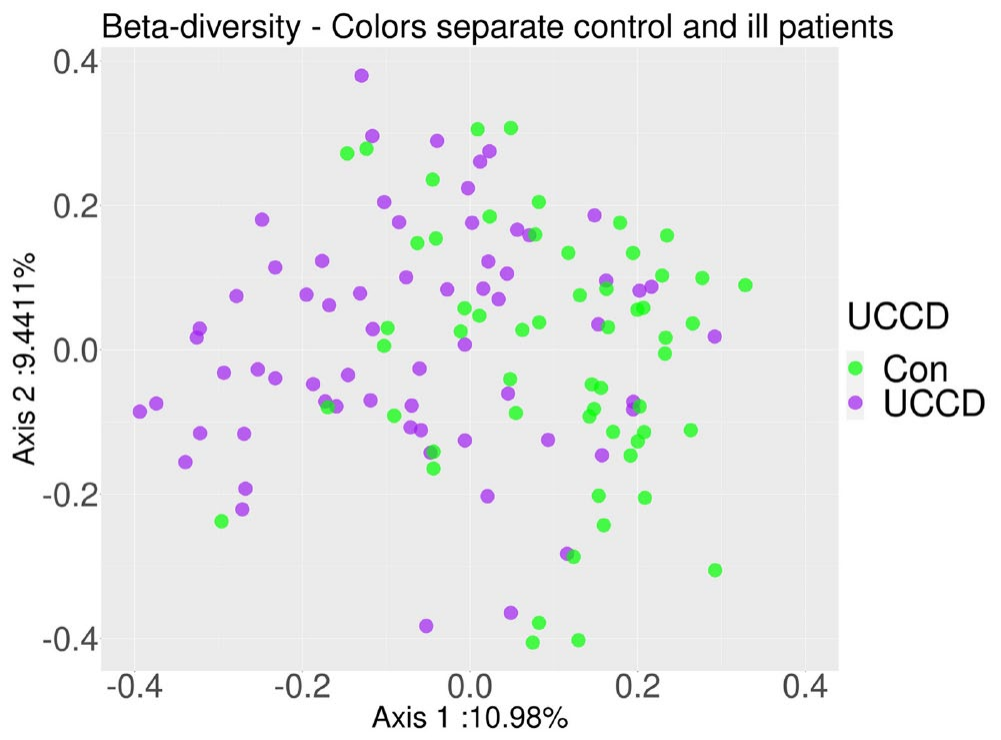
Principal coordinate analysis (PCoA) of beta diversity comparison of inflammatory bowel disease (IBD) patients and healthy (Con) participants. On the axes, the eigenvalues are shown as the percentage of the variation they explain.

The beta diversity values of the bacterial families differed markedly between the IBD and Con groups throughout the study. PCoA showed separated groups (*p* = 0.001) (Figure 5). From timepoint A to timepoint B, beta diversity of the Con groups changed the most (UC *versus* Con *p* = 0.03, CD *versus* Con *p* = 0.01, UC *versus* CD *p* = 0.58). The distance of UC to Con at timepoint C was higher than that at timepoint B; however, the statistical significance was marginal (*p* = 0.06). Other beta diversity comparisons did not show any significant changes. Coupling data with other comparison data are presented in Table 3 and Supplemental Figures.

**Table 3.** Beta (Bray–Curtis) diversity comparison between groups.

Comparisons	1st	2nd	*p* Value
UCA – UCB *versus* CDA – CDB	0.5	0.49	0.58
UCA – UCB *versus* Con-A – Con-B	0.5	0.53	0.03
CDA – CDB *versus* Con-A – Con-B	0.49	0.53	0.03
UCA – UCC *versus* CDA – CDC	0.53	0.48	0.01
UCA – UCC *versus* Con-A – Con-C	0.49	0.52	0.2
CDA – CDC *versus* Con-A – Con-C	0.48	0.52	0.11
UCA – Con-A *versus* UCB – Con-B	0.53	0.52	0.4
UCA – Con-A *versus* UCC – Con-C	0.53	0.55	0.26
UCB – Con-B *versus* UCC – Con-C	0.52	0.55	0.06
CDA – Con-A *versus* CDB – Con-B	0.55	0.55	0.45
CDA – Con-A *versus* CDC – Con-C	0.55	0.56	0.66
CDB – Con-B *versus* CDC – Con-C	0.55	0.56	0.19

1st and 2nd, the distances of first and second samples; A, B, and C, the time of sampling; CD, Crohn’s disease; Con, control group; UC, ulcerative colitis.

### Differential abundance analysis at the family level

Differential abundance analysis showed changes in numerous bacterial families of the examined groups. *Clostridiaceae* was found with higher abundance in the Con participants during the whole examination, and the abundances of *Brucellaceae*, *Moraxellaceae*, and *Alcaligenaceae* increased from timepoint A to B. Both of the IBD groups contained lesser abundance of the taxa *Pseudomonadaceae* than the Con group at timepoints B and C. The abundance of *Streptococcaceae* was higher in CD group at timepoint B compared to the Con group. Based on the differential abundance analysis, the abundance of *Clostridiaceae* and *Pseudomonadaceae* families increased, whereas that of *Bifidobacteriaceae*, *Carnobacteriaceae*, *Veillonellaceae*, and *Pasteurellaceae* families decreased in the CD group as compared to the Con group at timepoint B.

Three patients experienced a clinical relapse supported by a biochemical activity after lavage. Their samples were characterized by decreased abundances of *Bifidobacterium* and *Lactococcus* genera, whereas the frequency values of *Enterococcaceae* and *Streptococcaceae* families were increased after the bowel preparation.

Other less obvious changes in the bacterial family abundances are shown in the Supplemental Figures.

## Discussion

Our study is the first to investigate the effect of split-dose colon cleansing on the microbiome in CD and UC using a healthy control group at two timepoints after colonoscopy preparation in a relatively large patient population. Strict exclusion criteria were used to ensure that changes in gut flora were independent of other drug effects and that control results were not influenced by any pathological colonic abnormalities.

Our results showed statistically similar abundances of the examined group at the family level at baseline. The alpha diversity of the CD and Con groups did not change from timepoint A to timepoint C; however, the abundance of CD immediately after the bowel preparation (to timepoint B) was decreased. Beta diversity difference comparisons revealed the most flexible microbial changeability in Con participants; however, significant changes in the UC group were also prominent. The families of *Clostridiaceae* and *Pseudomonadaceae* had lower abundance in both IBD groups than in the Con group, whereas our results showed decreased *Bifidobacteriaceae*, *Carnobacteriaceae*, *Veillonellaceae*, and *Pasteurellaceae* and increased *Streptococcaceae* abundances in the CD group after the lavage.

IBD is a huge healthcare challenge in the field of gastroenterology. The presently used modern medical treatment and tight disease control of IBD are not generally successful due to conflicting etiology.^8,9^ Accordingly, the gut microbiota has been suggested as the potential causative agent of disease relapse and may affect the therapeutic efficacy, as it is clear that certain microbiome and metabolome abnormalities are associated with reduced therapeutic efficacy with different biological therapeutic agents. A pediatric IBD population-based analysis showed a higher baseline *Bifidobacterium* abundance in anti-tumour-necrosis-factor (anti-TNF) responder group than in the non-responder group.^29^*Roseburia* and *Burkholderia* species could predict the successful anti-integrin treatment in IBD patients according to a cohort study involving 85 CD and UC patients.^30^ An increased number of *Faecalibacterium* and *Bacteroides* species and decreased abundance of intestinal microbiota were associated with a higher likelihood of successful anti-TNF treatment.^31^ It is interesting to note that therapeutic modifications after colonoscopy are influenced by microbiota alterations as a result of bowel preparations. In our study, decreased *Bifidobacteriaceae* and increased *Streptococcaceae* abundances in the CD group after colonic lavage could be associated with lower therapeutic response. Furthermore, the success of the initiation of a new biological treatment after colonoscopy is dramatically influenced by the composition of the altered colonic microbiota based on the abovementioned results.

Only a few studies have analyzed the possible effect of bowel preparation on colonic microbiota especially in the IBD population. In the study of Drago *et al*., the gastrointestinal microbiota of healthy individuals was examined after a high-volume polyethylene glycol preparation. Based on their results, the preparation had a long-term effect on the microbiota composition and homeostasis, significantly reducing the number of protective bacteria and *Lactobacillaceae* abundance.^32^ Another study found short-term changes in the composition and diversity of gastrointestinal tract and fecal microbiota in healthy individuals and IBD patients after intestinal preparation. These alterations were more significant among the IBD patients.^15^ A prospective cohort study compared the alteration of gut microbiota composition after bowel preparation prior to colonoscopy using polyethylene glycol or bysacody in healthy participants and concluded a decreased long-term effect of the intestinal composition for most patients .^17^ Jalanka *et al.*^16^ found that majority of the intestinal microbiota recovered at 14 days after bowel cleansing using a preparation mixture based on polyethylene glycol. They also found that the rate of the recovery was dose dependent, and single-dose administration was associated with more severe effects on the gut microbiota.

Shobar *et al.*^15^ examined in a smaller cohort the microbiota-modifying impact of bowel preparation in both IBD patients and controls (*n* = 18). The results showed higher short-term changes in the disease group and raised the significance of two different luminal and mucosal microbial compartments of the gut.

Few experimental studies have been reported the effect of colonic lavage on gut microbiota in other systemic diseases. Chen *et al.*^33^ investigated the effect of split-dose colonic lavage with sodium sulfate among overweight male adults and concluded that the intestinal microbiota significantly differed at 7 and 28 days after lavage. Other examination among Parkinson’s disease patients focused on the effect of vegetarian diet and colonic enema on motor’s symptoms improvement. In the analysis reduced *Clostridiaceae* abundances were found after the oil-based enema compared to other groups which observation is contradicting with our data.^34^ The effect of enema raises the background mechanisms of microbiota alterations due to diarrhea or mechanic lavage. Bucher *et al.*^35^ in an experimental study revealed the significant loss of superficial mucus and epithelial cells which would be associated with altered bacterial composition.

The alpha diversity after the bowel preparation was the lowest in the CD group in our population, which is consistent with the findings of the available literature on CD pathogenesis.^36,37^

In general, the diversity of IBD patients’ gut microbiome showed a general decrease as compared to the healthy individuals’ gut, along with phylum-level decreases in *Firmicutes* and increases in *Proteobacteria*.^30^ In our study, we found increased *Clostridiaceae* and *Pseudomonadaceae* and decreased *Bifidobacteriaceae*, *Carnobacteriaceae*, *Veillonellaceae*, and *Pasteurellaceae* species in the CD group as compared to the Con group. In other studies on CD, the proportions of the *Clostridia* class were altered: the *Roseburia* and *Faecalibacterium* genera of the *Lachnospiraceae* and *Ruminococcaceae* families were decreased, whereas that of *Ruminococcus gnavus* increased.^8,9,30^ Based on our population, the healthy participants’ microbiota composition contained a higher level of *Clostridiaceae* species at each timepoint. The decreased abundance of *Pseudomonadaceae* in both IBD groups is also in line with the findings of previous literature.^8,9^

The effect of bowel cleansing on disease activity was revealed in our study. CRP was mildly elevated at baseline, which did not significantly change after the lavage; however, three patients experienced a relapse with an elevated CRP level. CDAI scores displayed remission in both measurement points, whereas the pMayo scores showed a mild disease activity. For this reason, a clear association between bowel preparation and disease flare was not proved; however, our results are limited owing to a small number of enrolled participants. The microbiota profile of patients with flare after the lavage in our cohort indicates the possible impact of *Lactobacillaceae* and *Bifidobacteriaceae* in IBD pathogenesis. Proliferation of opportunistic pathogens of the *Enterobacteriaceae* family could explain the flare-ups in IBD, as seen in our study. No previous study has analyzed the direct link of disease activity and bowel preparation; however, microbial alterations causing disease flares have been proven.^29–31,37^ It is notable, that the present study setting was not able to conclude clear causative relation of mechanism of microbiota alteration due to special agent of bowel preparation and disease flare after bowel preparation, but it raises the possibility of that. In this way, further controlled studies with different types of laxatives and larger number of enrolled participants are needed to determine the mechanical or agent-specific biological effect of bowel cleansing on gut microbiota.

There are several factors that limit the interpretability of the results of our current study. A total of 41 participants were enrolled, which is below the number of patients ideally expected. Thus, a subgroup analysis to exclude interfering distortion was not possible. It is notable that 16S rRNA sequencing is still the most widely used method in microbial analyses, although it does not provide the most accurate results when comparing the accuracy of its results to the resolution of the shotgun sequencing technique. The study design made it impossible to differentiate the luminal and mucosal microbial changes. Our study focused on the split-dose usage of sodium picosulfate; however, other laxatives may have contradicting effects on intestinal microbiota. Furthermore, viral and fungal components of the gut microbiota and metabolites produced by the intestinal organisms were also not analyzed in the present study. It needs to be mentioned that intestinal microbiota is altered with aging,^38,39^ and in our cohort, the UC patients were older than the other two groups.

Despite the abovementioned limitations, our study clearly adds to the very limited literature available in the patient population examined. One study strength was that our data represent real-life situations with exactly defined study groups. Simple comparisons provide a relatively low amount of type I errors compared to multiple testing. Microbial analytic methodology used in recent study follows the commonly used protocols, allowing the reproducibility and comparability of our results found. A higher number of enrolled participants and a longer follow-up period with several sampling points would allow us to obtain more accurate data; however, the cost of the analysis would be increased. It should be highlighted that our 41 enrolled participants are below the expected to conclude strength implications; however, the literature available in this topic reports data of studies investigating the same magnitude of enrolled participants (*n* = 10–18).

In conclusion, our findings suggest that bowel preparation can result in a significant change in fecal microbial composition in IBD. Microbial alterations recovered earlier in UC patients. Reduced alpha diversity and altered abundance in CD may have a potential role in disease exacerbation and may decrease the efficacy of a newly started biological. Moreover, we suppose that microbial composition shifting can modify the microenvironment in the colon, which can determinate disease activity. Furthermore, we propose to define the indication of the colonoscopy exactly to avoid the occurrence of possible flare-ups due to the altered microbiota. It could be suggested to highlight the importance of timing of colonoscopy considering further therapeutic modifications and possible disease flares. Further studies are needed to verify our data and to establish possible therapeutic suggestions and consequences and to specify the best bowel preparation regimen and substance in case of IBD patients.

## Supplemental Material

sj-docx-1-tag-10.1177_17562848231174298 – Supplemental material for Effects of bowel cleansing on the composition of the gut microbiota in inflammatory bowel disease patients and healthy controls Supplemental material, sj-docx-1-tag-10.1177_17562848231174298 for Effects of bowel cleansing on the composition of the gut microbiota in inflammatory bowel disease patients and healthy controls by Péter Bacsur, Mariann Rutka, András Asbóth, Tamás Resál, Kata Szántó, Boldizsár Jójárt, Anita Bálint, Eszter Ari, Walliyulahi Ajibola, Bálint Kintses, Tamás Fehér, Daniella Pigniczki, Renáta Bor, Anna Fábián, József Maléth, Zoltán Szepes, Klaudia Farkas and Tamás Molnár in Therapeutic Advances in Gastroenterology

## Declarations

**Ethics approval and consent to participate:** The present study was approved by the Regional and Institutional Human Medical Biological Research Ethics Committee, University of Szeged (approval no.: 51/2019-SZTE) and carried out according to the guidelines stipulated in the Declaration of Helsinki. All patients provided written informed consent prior to study participation.

**Consent for publication:** TM is the guarantor of the article. All authors have read and agreed to the submitted version of the manuscript.

**Availability of data and materials:** The data cannot be shared publicly for the privacy of individuals that participated in the study. The data underlying this article will be shared on reasonable request to the corresponding author. The current manuscript, including related data and figures, has not been previously published and is not under consideration elsewhere.
