# Supplementary material for: Effects of bowel cleansing on the composition of the gut microbiota in inflammatory bowel disease patients and healthy controls
Source: Ther Adv Gastroenterol. 2023 Jun 6;16:17562848231174298. doi: 10.1177/17562848231174298 (PMC10265323; doi:10.1177/17562848231174298)
Supplement: sj-docx-1-tag-10.1177_17562848231174298 – Supplemental material for Effects of bowel cleansing on the composition of the gut microbiota in inflammatory bowel disease patients and healthy controls [file sj-docx-1-tag-10.1177_17562848231174298.docx]

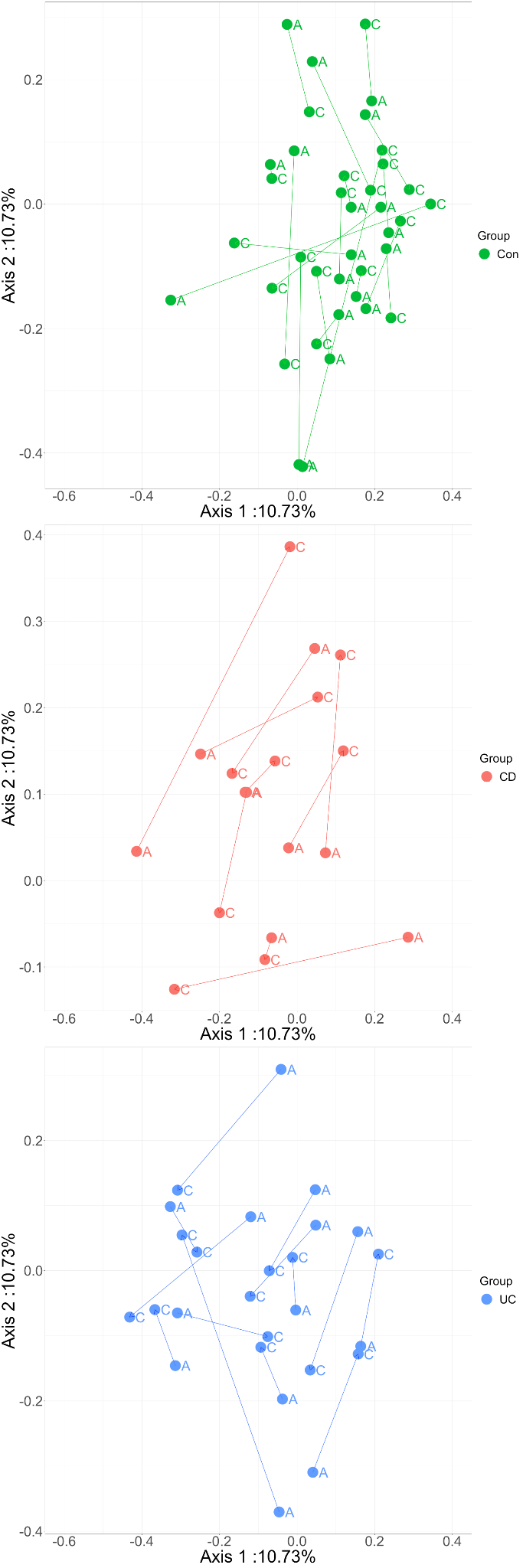


**S Figure 1.** Beta distance diversities of samples between timepoints A and C in the respective groups: Con CD and UC visualized on a PCoA plot. The labels on the axes represent the eigenvalues.


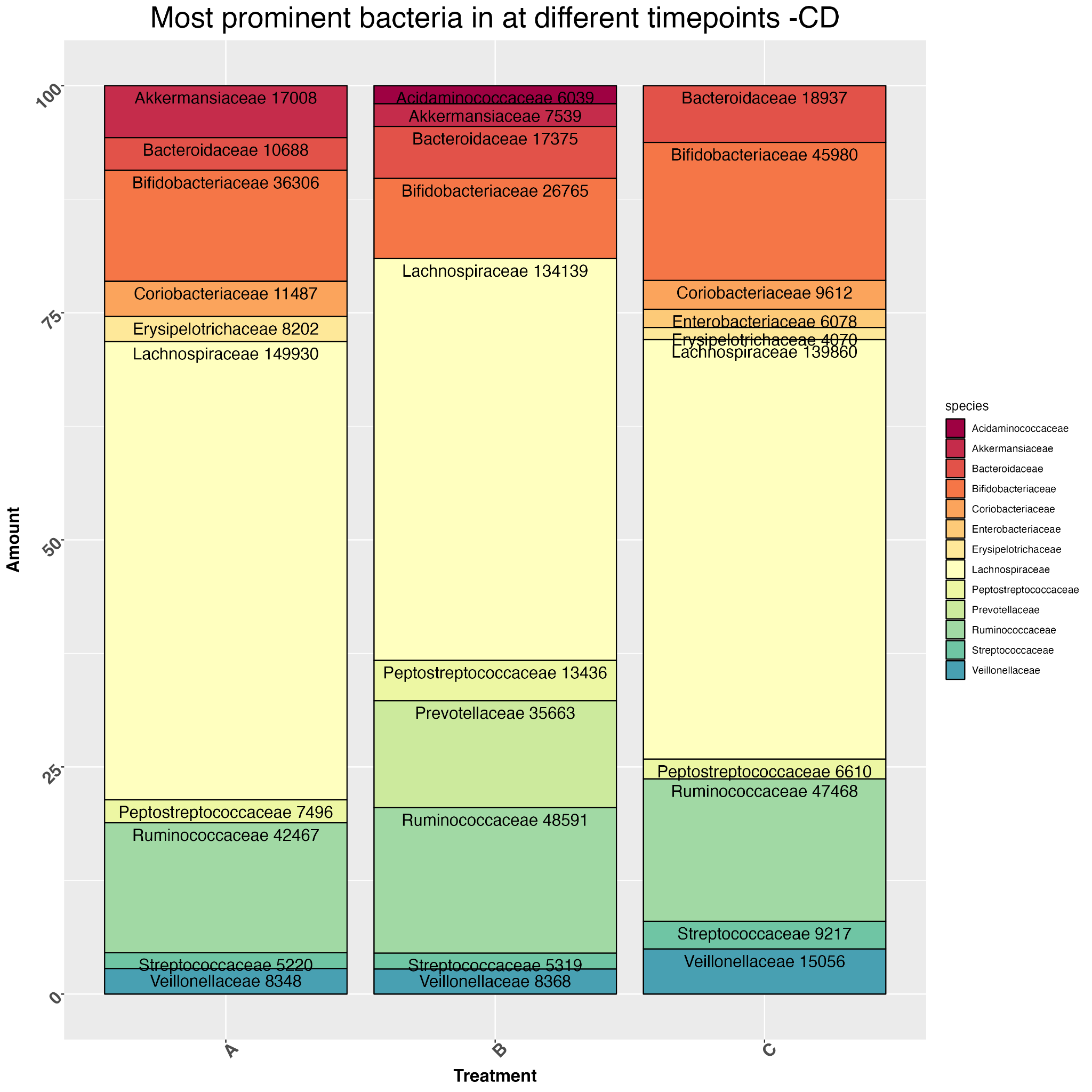


**S Figure 2.** Most abundant bacterial families in the different timepoints in the CD group.


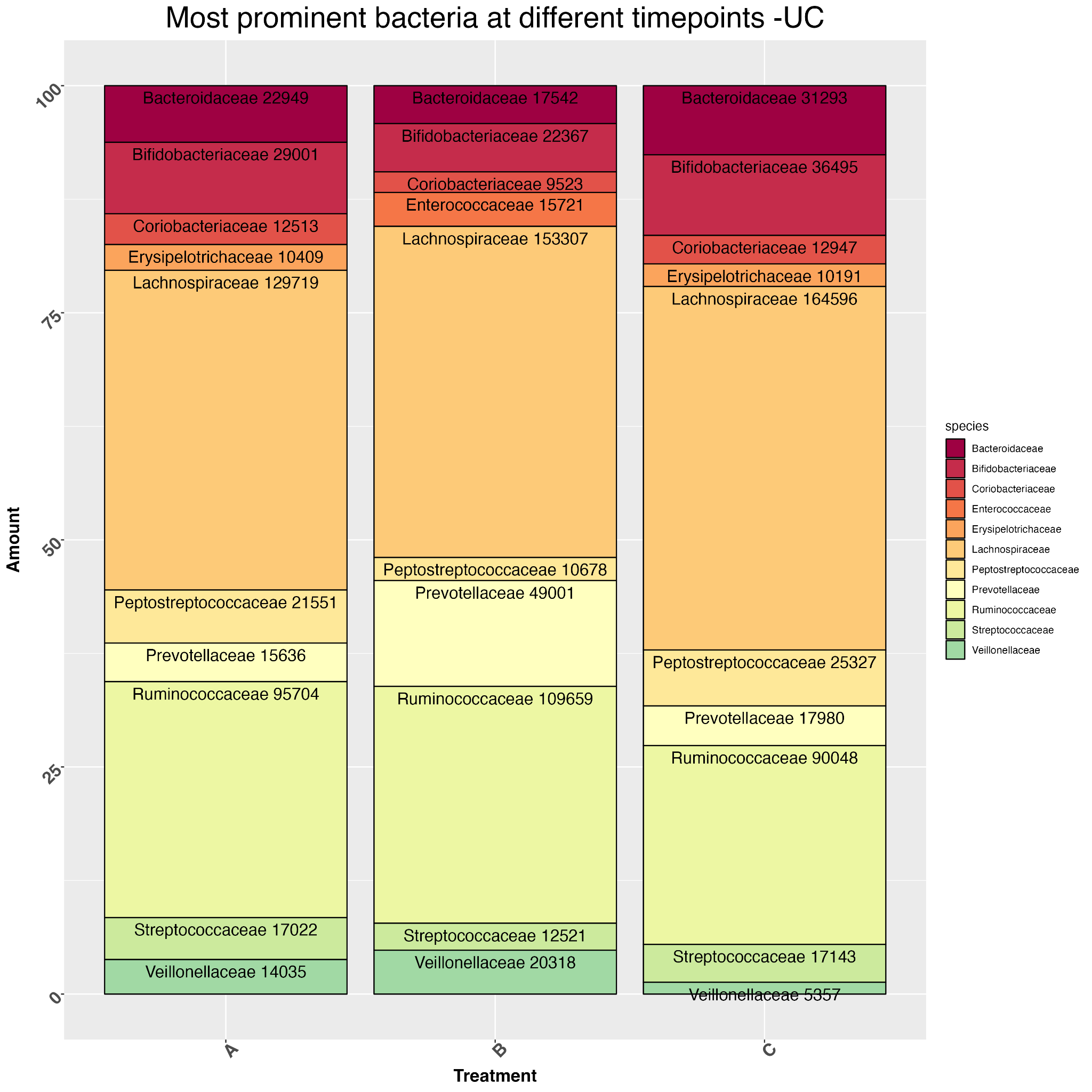


**S Figure 3.** Most prominent bacterial families in the different timepoints in the UC group.


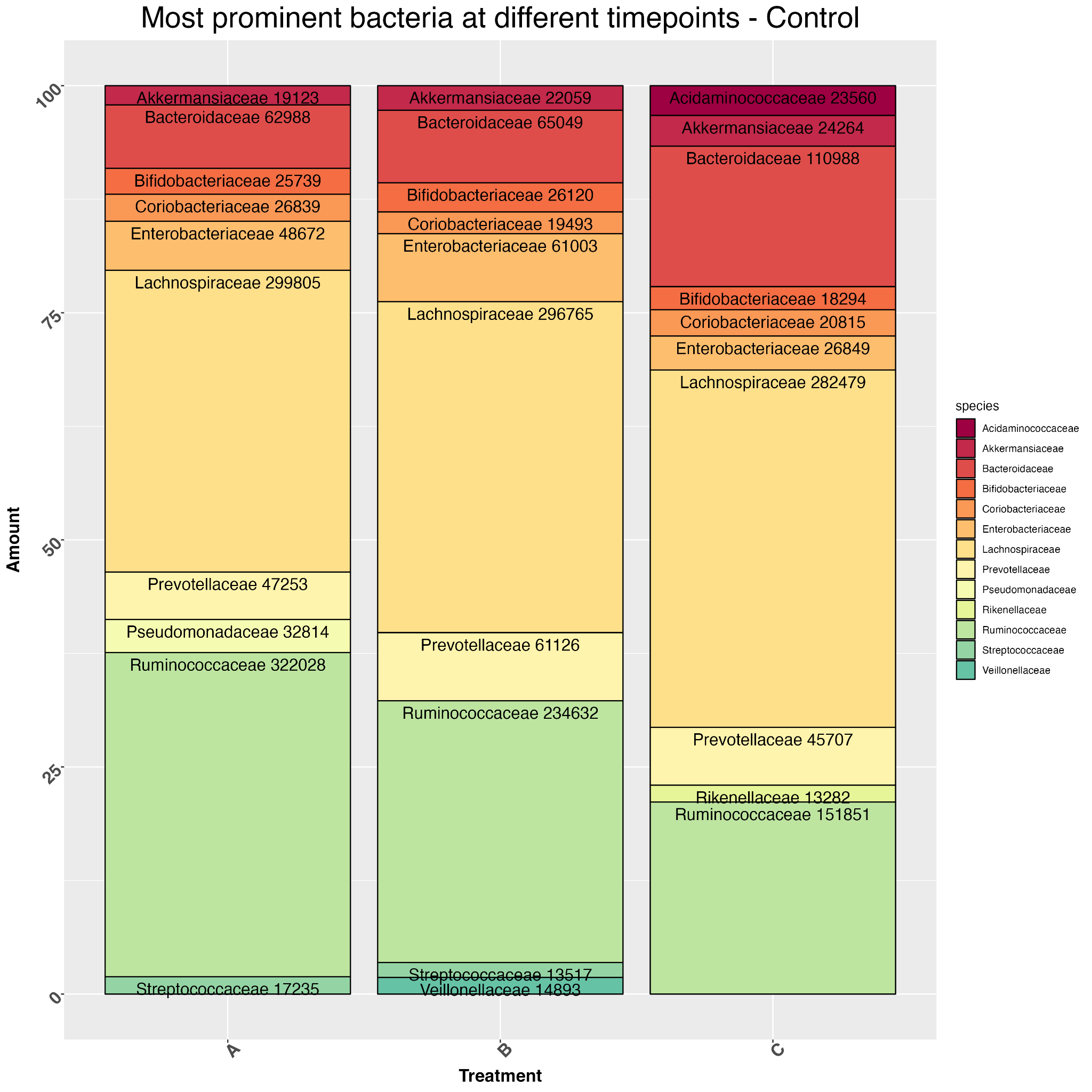


**S Figure 4.** Most prominent bacterial families in the different timepoints in the control (Con) group.


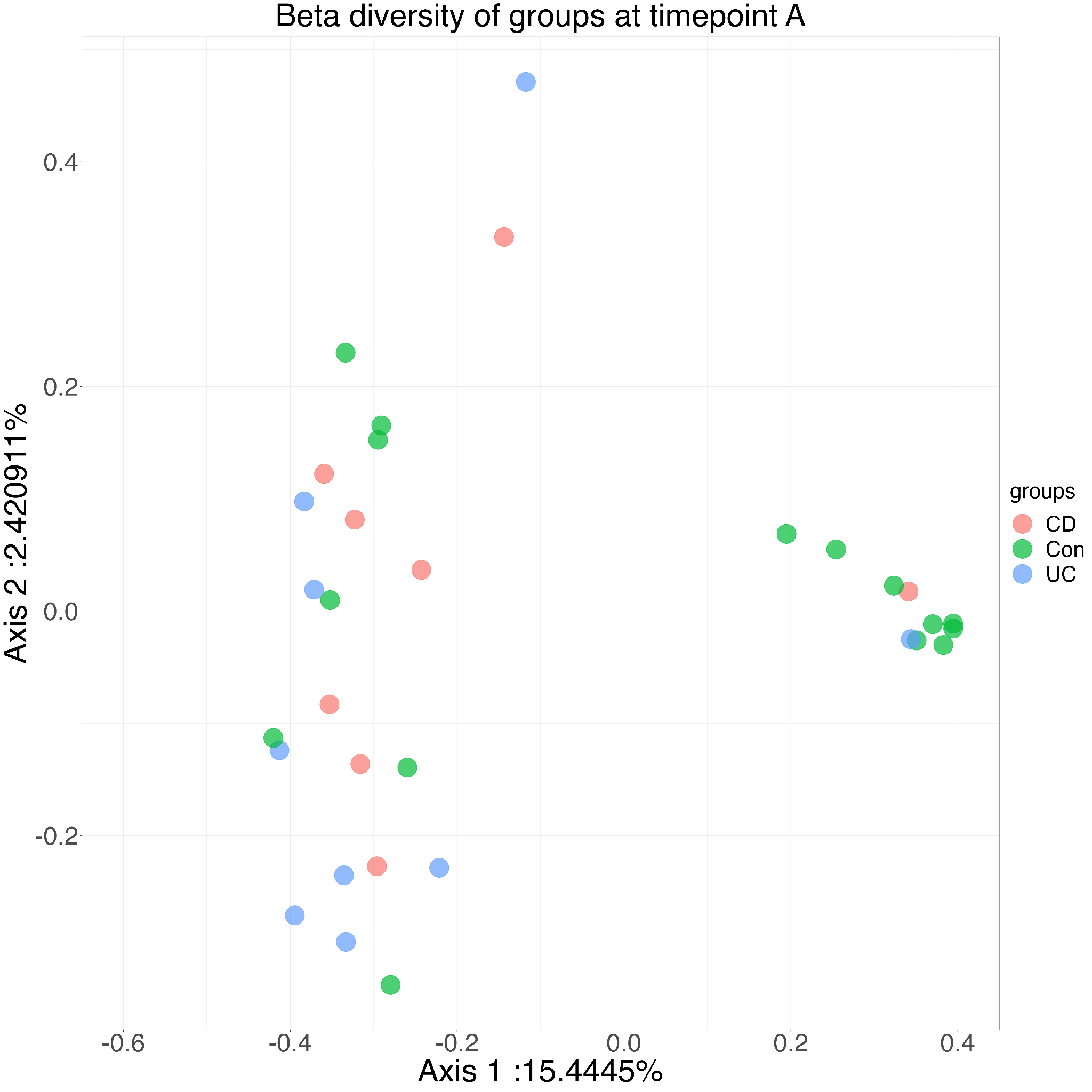


**S Figure 5.** Beta diversities of samples of each group in timepoint A visualized on a PCoA plot. The labels on the axes represent the eigenvalues.


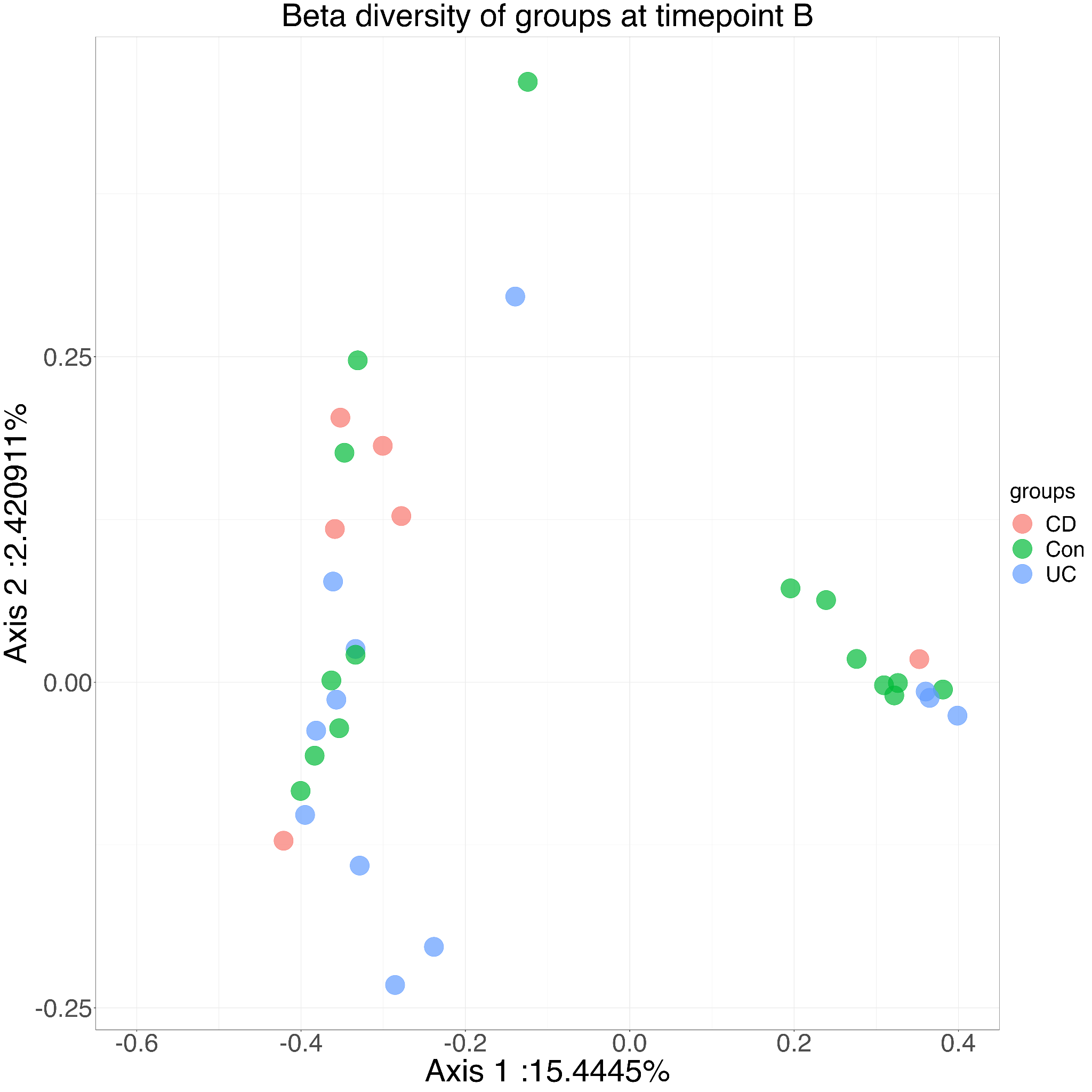


**S Figure 6.** Beta diversities of samples of each group in timepoint B visualized on a PCoA plot. The labels on the axes represent the eigenvalues.


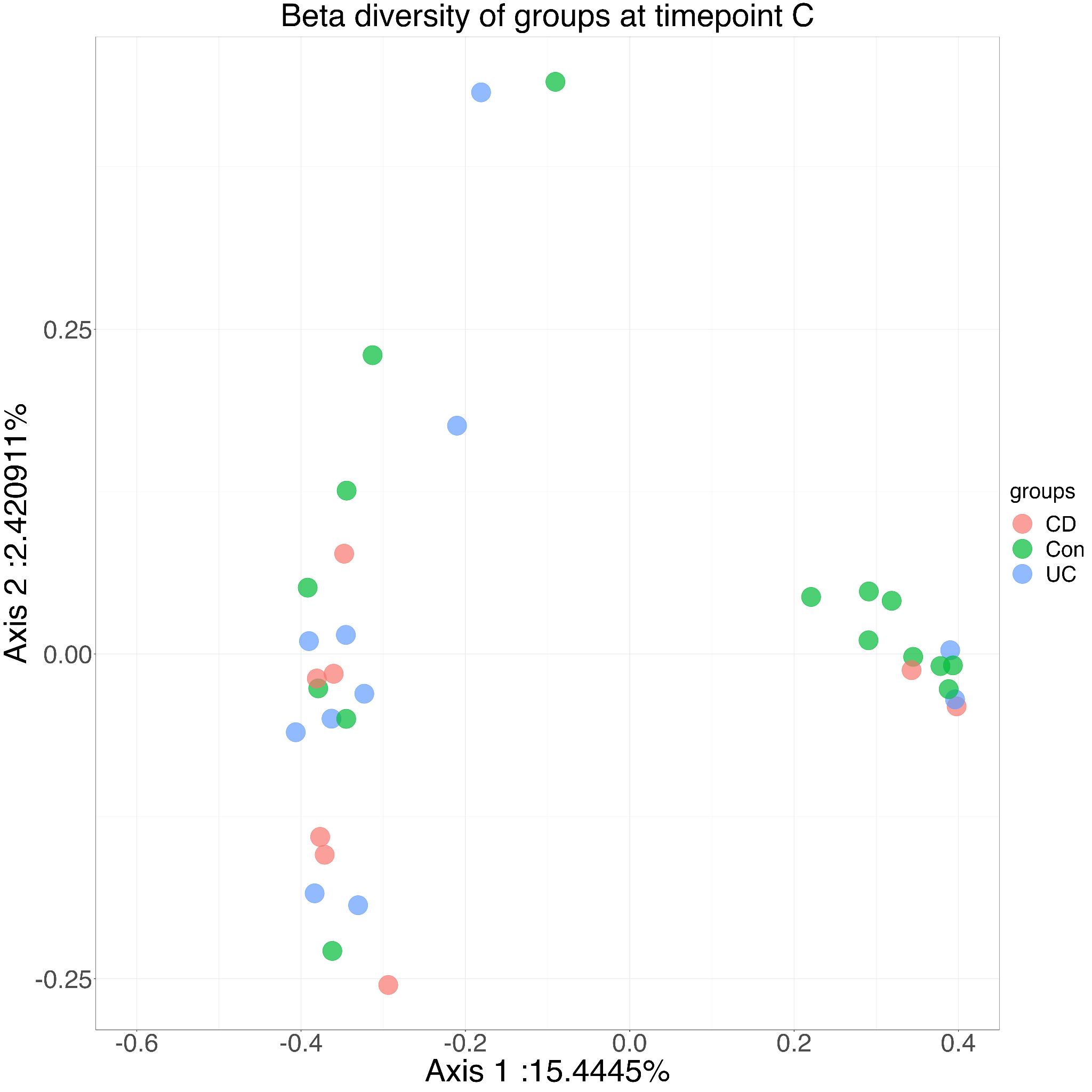


**S Figure 7.** Beta diversities of samples of each group in timepoint C visualized on a PCoA plot. The labels on the axes represent the eigenvalues.


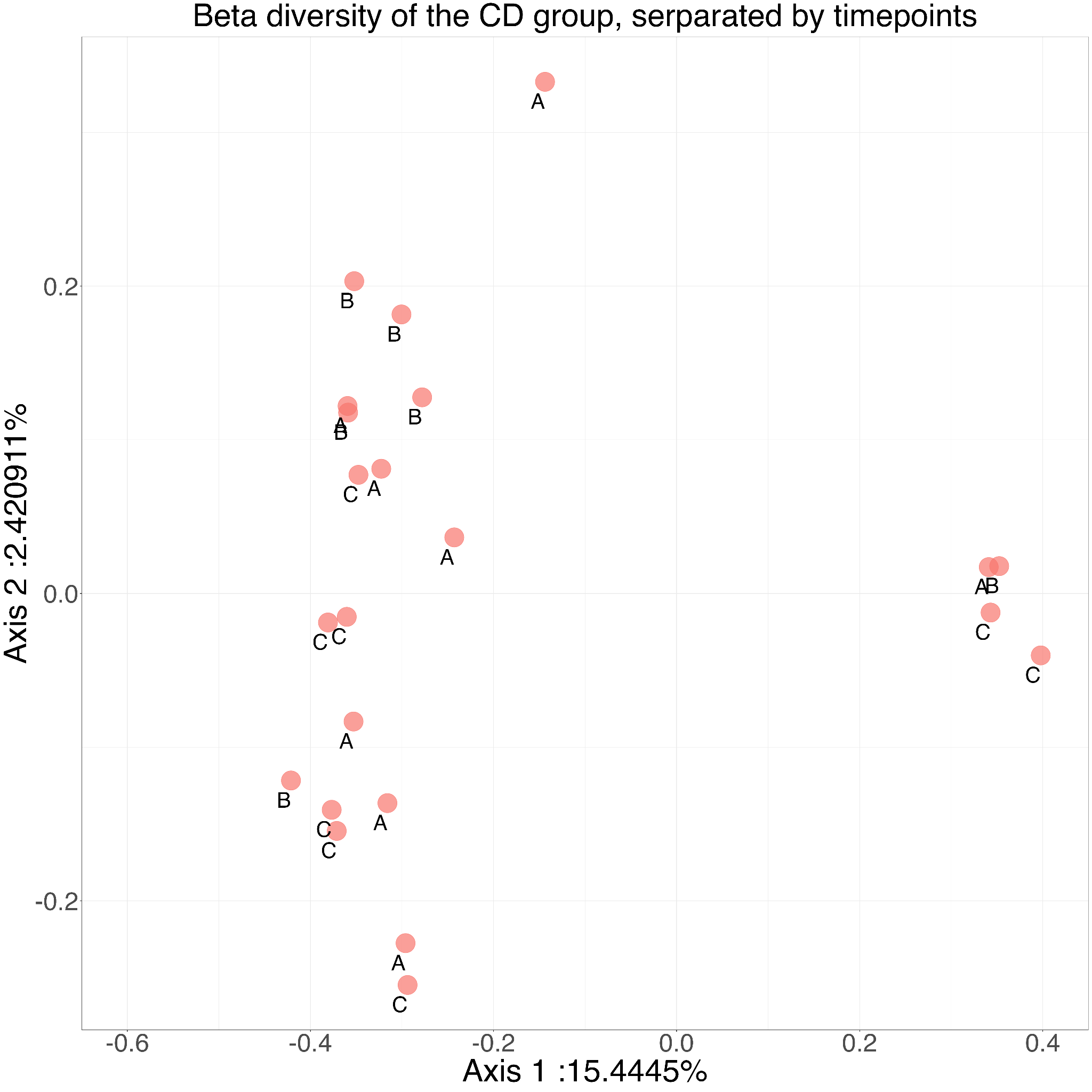


**S Figure 8.** Beta diversities of samples between timepoints A and C in the CD group visualized on a PCoA plot. The labels on the axes represent the eigenvalues.


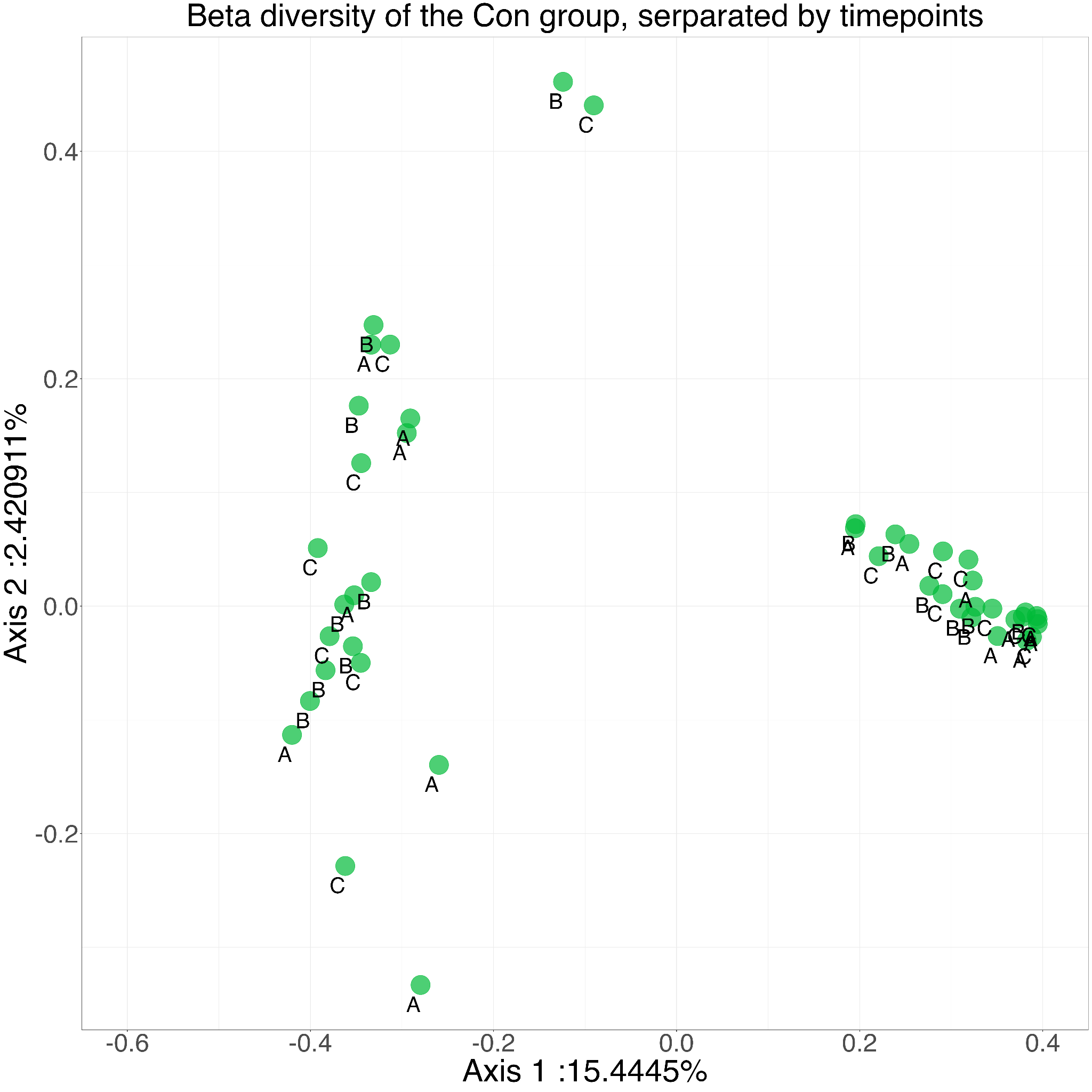


**S Figure 9.** Beta diversities of samples between timepoints A and C in the Con group visualized on a PCoA plot. The labels on the axes represent the eigenvalues.


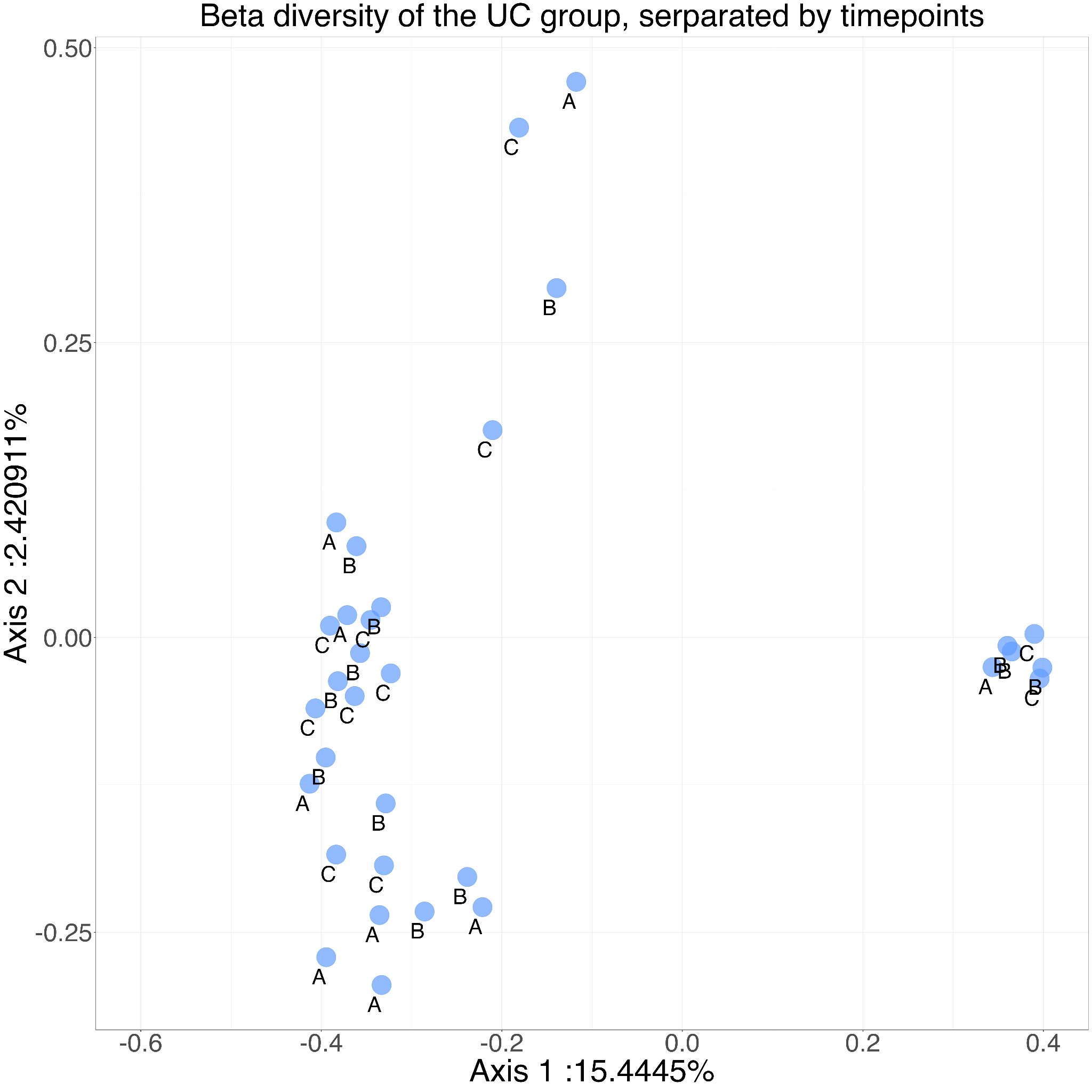
 **S Figure 10.** Beta diversities of samples between timepoints A and C in the UC group visualized on a PCoA plot. The labels on the axes represent the eigenvalues.

**Power analysis**

The power analysis test can be used to determine the sample size needed for a statistical test to detect deviation from the null hypothesis given a required significance level, and effect size. To provide an appropriate visualization of this relationship we did test simulations with growing effect sizes, in our case growing relative differences between the samples.

For guidance on how to simulate the data we looked into the spread of values in the control (Con) group, which (aside from some outliers) showed a normal distribution. We measured the mean and the standard deviation to be 0.5 and 0.16 respectively (Figure 1). We conducted the same experiment for Alpha-diversity values and found these numbers to be 1.76 and 0.33 (Figure 2). Using these parameters we have generated simulated values for each sample groups (CD, UC, and Con) and tested them against each other to see if we receive a significant p-value. From 0 to 100 relative differences we increased the effect size by steps of 5, and measured how many of 1000 Mann Whitney U-Tests (the statistical test we’ve used in the manuscript) showed significant difference. The outputs of this process were the power analysis plots, showing the rate of detection we can expect with a certain effect size. These visualizations illustrate that with higher effect sizes detection of changes is more likely, and since CD has a lower sample size than the other two groups, it follows a lower trajectory (Figure 3 and 4).

Since lower sample size hinders the sensitivity of our method, it does not discredit its findings. Also, as described in the original paper, the Mann Whitney U test is valid with different sample sizes and does not produce any false results due to these differences.^^[[1]](#footnote-1)^^ The first question however correctly highlights that with larger datasets, the Mann Whitney U-test becomes sensitive to smaller biologically relevant changes, which the authors always strive to satisfy within the boundaries of availability.


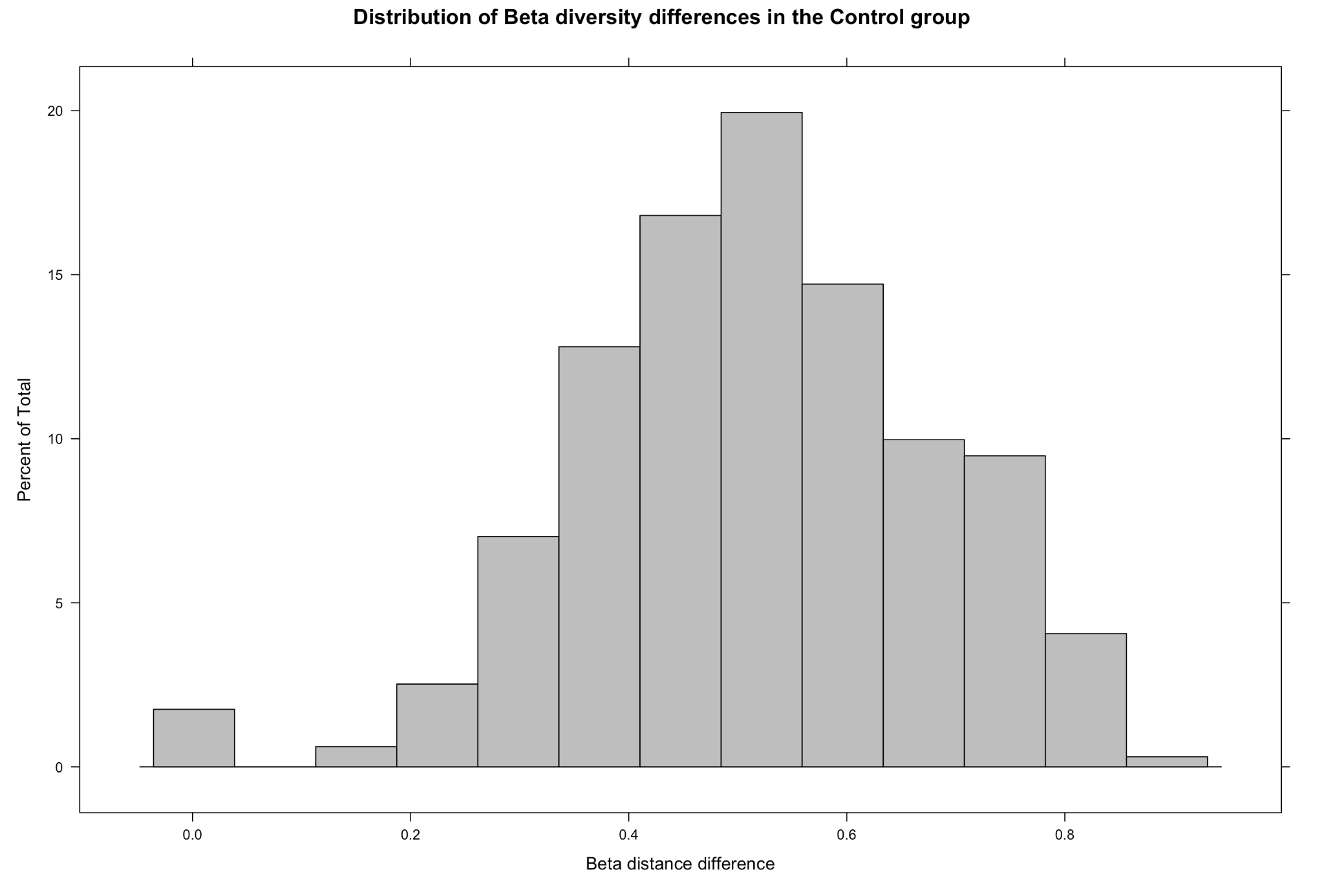


Figure 1 : Distribution of the Beta diversities in the control (Con) group, used for a basis of simulating the effect sizes and detection ratios.


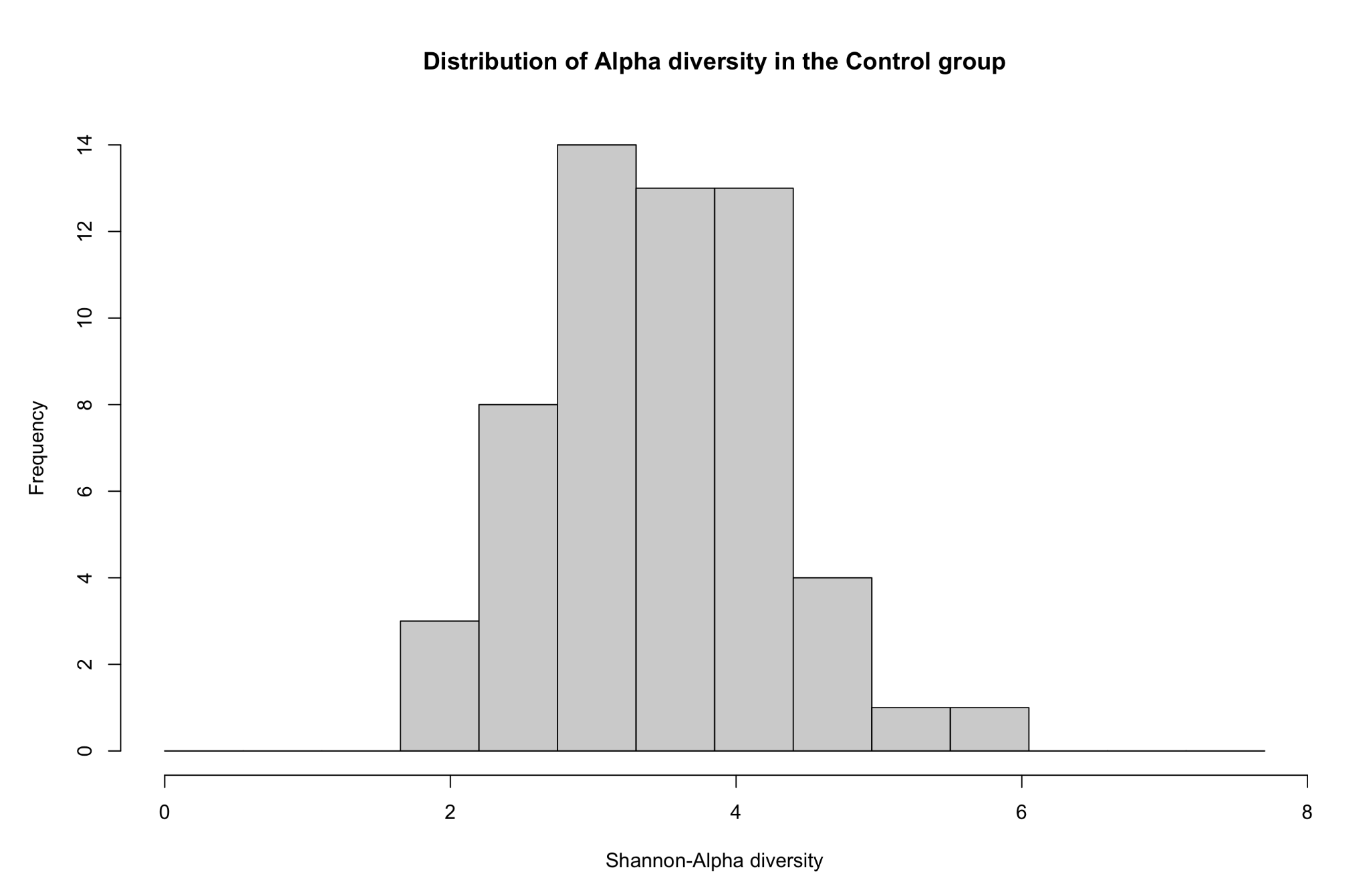


Figure 2: Distribution of the Alpha diversities in the control (Con) group, used for a basis of simulating the effect sizes and detection ratios.


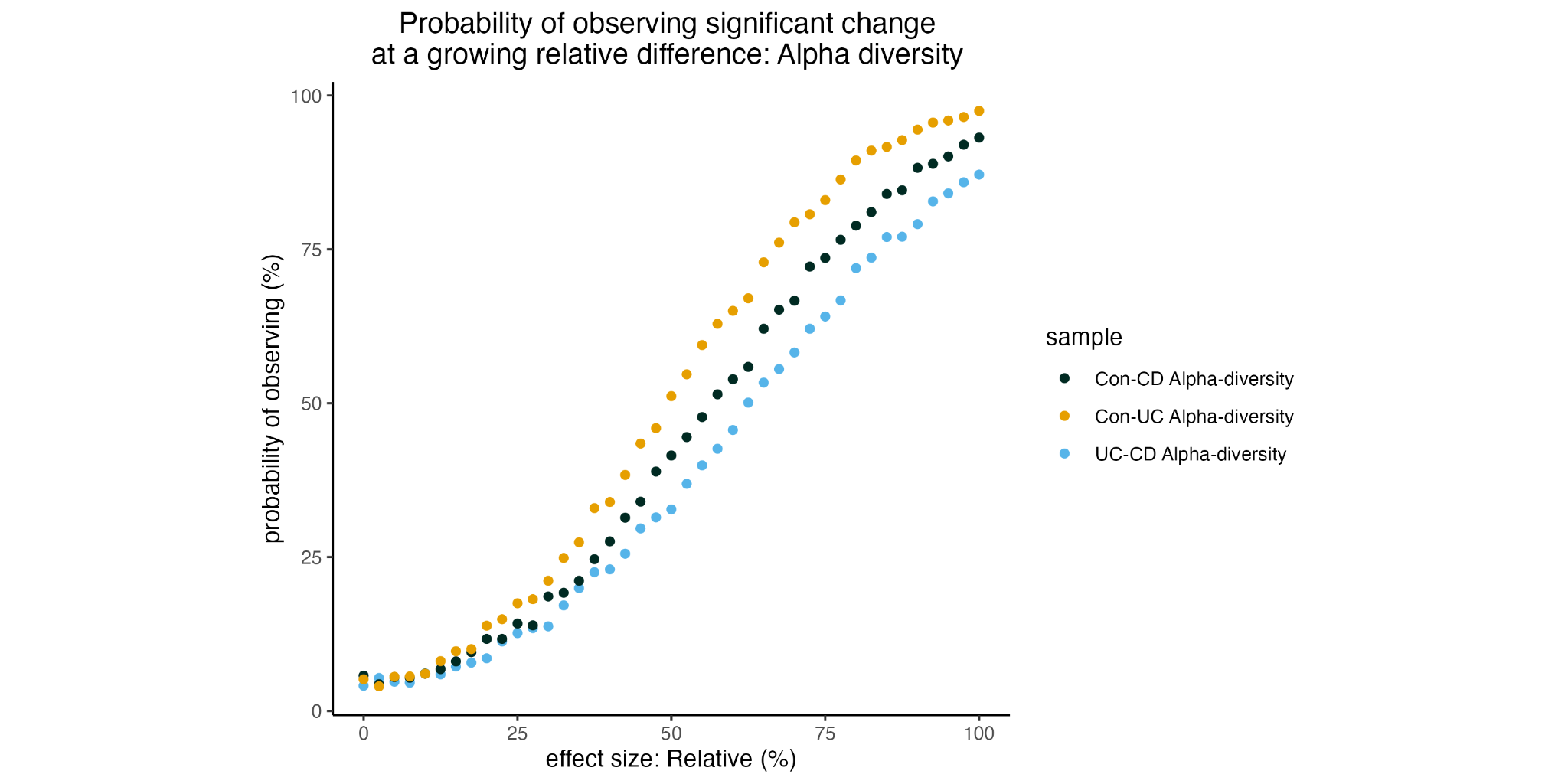


Figure 3: Probability of observing significant changes in Alpha diversity as the relative difference between the samples grows. As CD and UC have the lowest sample sizes, that comparison requires the largest effect to detect a significant change.


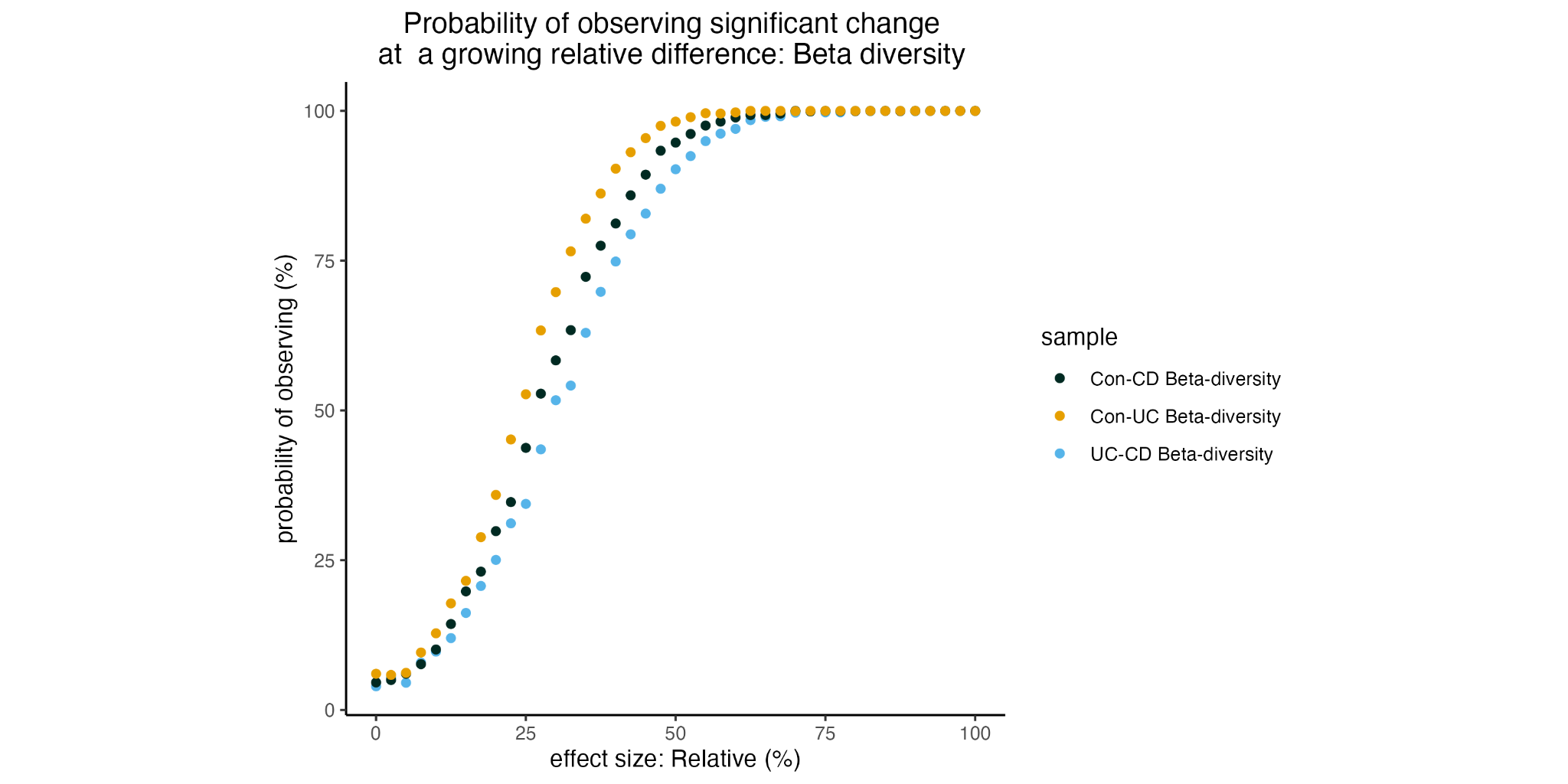


Figure 4: Probability of observing significant changes in Beta diversity as the relative difference between the samples grows. As CD and UC have the lowest sample sizes, that comparison requires the largest effect to detect a significant change.

Considering the previous findings, to show how sample size affects detection, we have redone all of the Mann Whitney U-tests by randomly sampling each group to be the same size as the smaller one in the comparison 1000 times, and counted how many times we received significant results (%). The results show that in case of smaller effects, had we used a smaller sample size of both groups, we would have had less chance of detecting the significant difference. We believe that this however does not invalidate the findings that we’ve had, again emphasizing that the Mann Whitney U-test does not produce false findings due to differences in the sample size of the two groups compared (Table 1 and 2).

Table 1: Comparisons of Beta diversities, signifying the respective means of the distances, their original p-values in the manuscript and how many times the significance was found (%), when we downsampled the larger group, to be the same size as the smaller one.


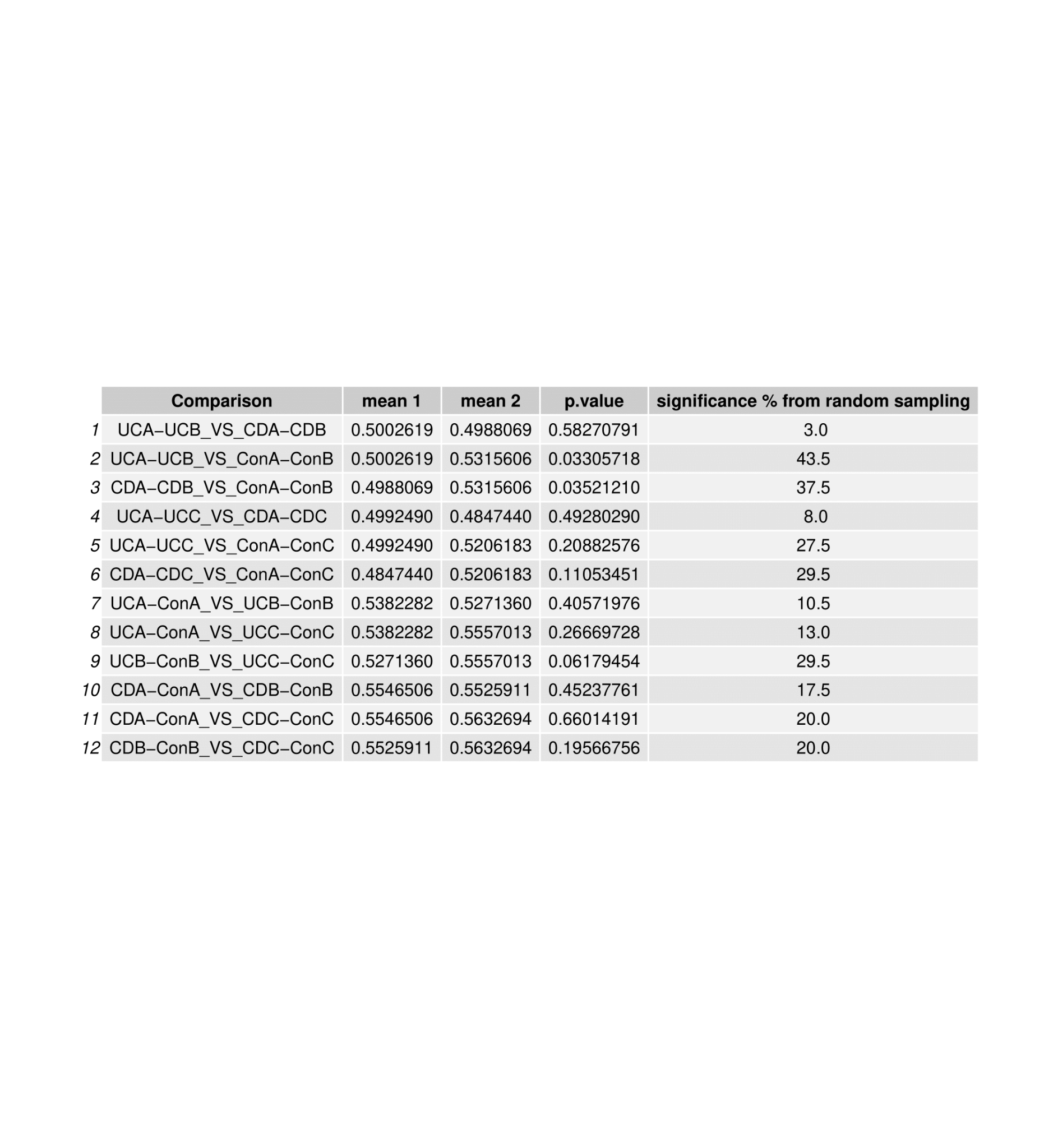


Table 2: Comparisons of Alpha diversities, signifying the respective means of the diversity values, their original p-values in the manuscript and how many times the significance was found (%), when we downsampled the larger group, to be the same size as the smaller one.


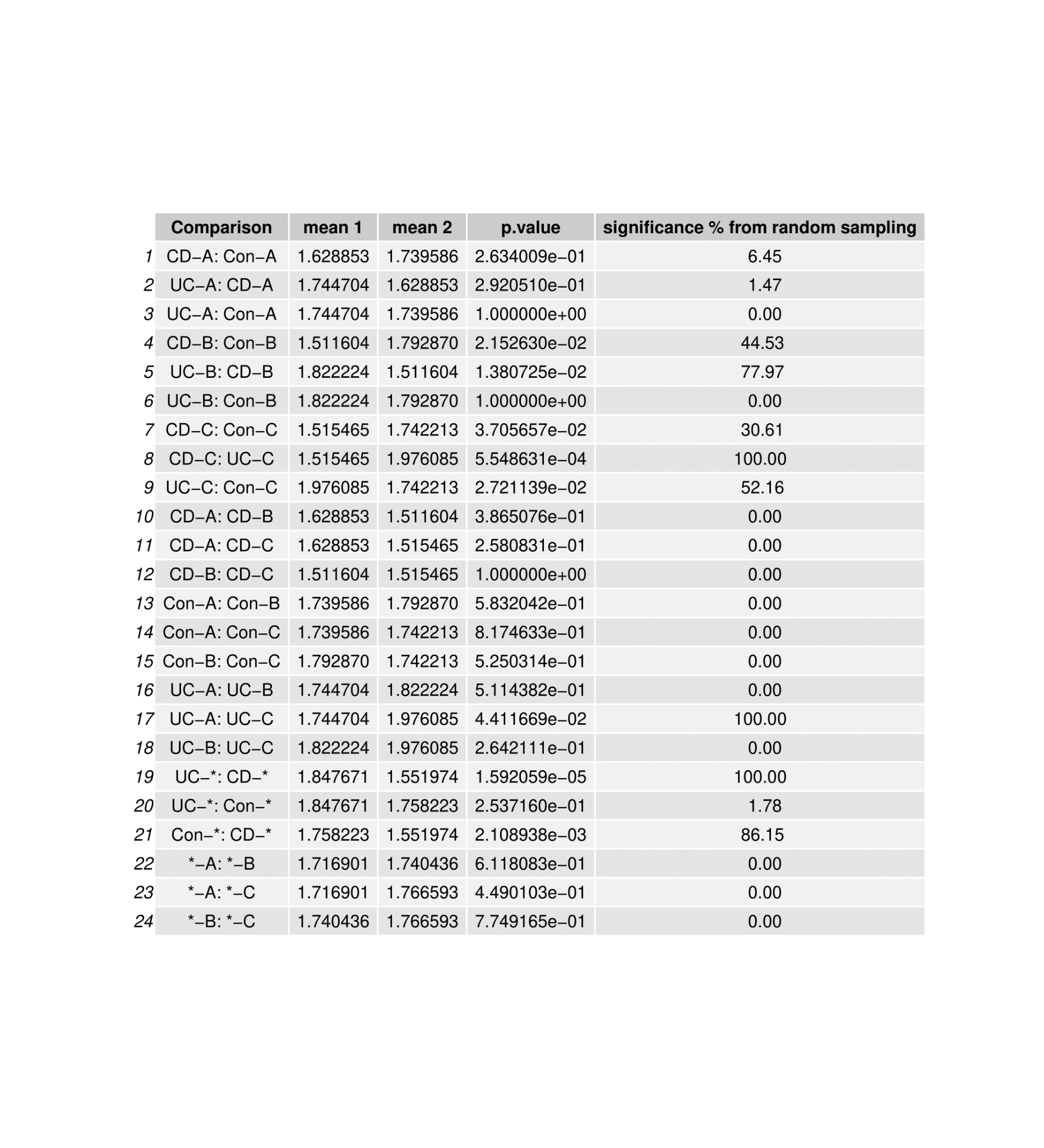


1. Mann, H.B. and Whitney, D.R. (1947) On a Test of Whether One of Two Random Variables Is Stochastically Larger than the Other. Annals of Mathematical Statistics, 18, 50-60. [↑](#footnote-ref-1)
